# Forensic Outpatient Variables That May Help to Prevent Further Detention

**DOI:** 10.3389/fpsyt.2020.00042

**Published:** 2020-02-13

**Authors:** Karoline Klinger, Thomas Ross, Jan Bulla

**Affiliations:** ^1^ Department of Forensic Psychiatry and Psychotherapy, Centre of Psychiatry, Reichenau, Germany; ^2^ Department of Psychosomatic Medicine and Psychotherapy, Ulm University, Ulm, Germany; ^3^ Department of Forensic Psychiatry and Psychotherapy, Ulm University, Günzburg, Germany

**Keywords:** forensic outpatient treatment, living conditions, desistance, violence, violent behaviour, forensic psychiatry

## Abstract

**Background:**

Forensic outpatient treatment in Germany helps forensic patients back into society while managing the risk that these individuals present to public safety. Measures used to achieve this objective include ongoing psychiatric treatment and monitoring, case management, and controlling risk factors that may cause criminal behavior. In addition to the effects of treatment and control, good living conditions have been hypothesized to help prevent criminal recidivism and a number of studies have examined variables related to poor outcomes including recidivism among former prison inmates and sexual offenders. Yet, little is known about the predictive validity of certain candidate variables on the outcomes of German forensic outpatients.

**Methods:**

In order to investigate variables that are likely to reduce the risk of unfavorable outcomes such as subsequent confinement or back-referral to inpatient treatment, we analyzed data from a forensic outpatient data project run by the federal state of Baden-Württemberg (Forensic outpatient documentation system). Based on data provided by six forensic treatment units throughout the federal state of Baden-Württemberg since 2015, we compared 61 forensic outpatients that had either regularly ended treatment (group one, n = 25), or were referred back to a forensic hospital or prison (group two, n = 36). Information on the patients' working, living, and financial situation as well as information on their social network and relationship status, was used. The predictive validity of these factors on treatment outcome was tested with a logistic regression model.

**Results:**

There were a number of *a priori* differences between the groups, but pro-social leisure activities in an outpatient environment and migration status were the only significant predictors of positive vs. negative outcome.

**Discussion:**

Implications of these findings are discussed.

## Introduction

The forensic aftercare system is expected to support patients in their attempt to live their lives without putting the society and themselves in danger (1, p. 12). Additionally, the outpatient system is thought to relieve forensic psychiatric facilities from growing patient numbers [e.g. (2)]. Several studies reported promising effects of specific forensic aftercare on relapse rates (see 3 for a review), but general and violent criminal offence recidivism are best predicted by eight central risk factors for offending in the general forensic population. These factors comprise criminal history, pro-criminal attitudes, antisocial personality pattern, pro-criminal peers, education/employment, family/marital stability and relationships, and substance abuse (for a substantial review see 4). A recent meta-analysis by Eisenberg and colleagues (5) confirmed the predictive value of the central eight risk domains for general and violent recidivism among forensic outpatients. The evidence gathered in this study is strong (22 studies were included, yielding 543 effect sizes in a population of nearly 117.000 adult offenders), but of course, there is other scientific evidence on (single) variables or factors associated with successful forensic aftercare in terms of desistance from criminal activity: substance abuse, housing, employment, interpersonal relationships and family support, and criminal involvement are all candidate factors determining the likelihood of favorable outcomes (6, p. 36 ff; 7).

*Substance abuse* has long been described as a core risk factor for (persisting) delinquency (8, 9). Assuming a direct causal relationship between drug use and criminal behavior, it is argued that desisting from drug use is a critical step towards desistance from crime (10). Social inclusion and identity change play an important role in moderating the relationship between substance abuse and crime (8).

Warr (11) hypothesized that *marriage* may be a core protective agent as being married changes a person´s social network and the time spent with (delinquent) peers. Being single or never being married were negatively related to successful competency restoration in a study about patient characteristics and outcomes with respect to successful outpatient competency to stand trial (12). In another recent study, Forrest (13) focused on the role of cohabitation and relationship quality in the empirically established link between marriage and desistance in the general population. They found no effect of mere cohabitation on delinquency, but marriage was associated with significantly lower ratios for violent delinquency, property delinquency, and drug delinquency. Importantly, this effect depended on the *quality of the relationship*, with better relationships yielding better protective effects on criminality at large. In a Dutch study on the quality of life of forensic patients with a personality disorder and patients with a mayor mental disorder, Bouman (14) reported that patients with a major mental disorder were less often in a relationship or had children; they less often had a job, enjoyed less social support, were hospitalized more often in a psychiatric hospital, but had fewer financial commitments and debts than the personality disorder group. Overall, the patients with a major mental disorder scored higher on a subjective quality of life rating than the personality disorder group. This indicates that there are meaningful differences between different groups of psychiatric patients regarding their living conditions and the ways of how these individuals perceive their quality of life.

Long-term outcomes on individuals with serious mental illnesses or psychiatric disabilities may depend on their *social placement* in the community. In a study of 91 men and women with severe co-occurring disabilities who had been acquitted of violent crimes by reason of insanity, Smith et al. (15) found that positive outcomes in terms of non-reoffending were associated with psychiatric stability, substance abuse abstinence, stable housing, and meaningful activity. Interestingly, they also found that individuals who lived with their families of origin showed the poorest overall success rate in terms of substance abstinence and housing stability. When mentally disordered offenders discharged from forensic psychiatric care are placed in socially disorganized neighborhoods (some of which may correspond with the neighborhoods the patients stem from and where their families of origin still live), there is evidence that their chance of returning to forensic psychiatric inpatient care may be elevated (16).

Nilsson and Estrada (17) reported a strong link between delinquency and the connectivity to the *labor market*, with unemployment negatively affecting delinquency rates from childhood into adulthood. Disconnection from the labor market fosters poor economic living conditions which in turn are associated with mental illness and offenses committed by mentally ill offenders (18). *Criminal involvement* during conditional release is related to involuntary readmission to a forensic hospital. In a study investigating factors associated with voluntary and involuntary readmissions to forensic hospitals, Marshall et al. (19) found that treatment non-compliance and arrests predicted involuntary admissions. Furthermore, low numbers of community psychiatric admissions and a longer duration in the community prior to any psychiatric readmission were associated with desistance, i.e. these individuals were less likely to be readmitted to forensic treatment while on conditional release.

Moreover, there might be gender differences in the occurrence and the effect of turning points on desistance. While the proportion of women committing crimes in general is substantially lower than in men, delinquent women often face even more *social exclusion* and *welfare deficiencies* than their male counterparts (17, 20). The quality and frequency of *social contacts*, in spite of limited social integration, may help forensic outpatients to better adjust to the challenges of community life (21).

Drawing on the literature presented above, the aim of the current study was to investigate factors that may meaningfully be related to the outcome of Baden-Württemberg forensic outpatient treatment. In the current study, outcome was defined positive when a therapy ended as planned by the treating team. It was negative when a patient recidivated, when the court revoked outpatient treatment, or re-hospitalization ensued. The main research idea was to identify variables associated with forensic outpatient treatment success.

In accordance with the literature our main hypothesis was that the quality of living conditions, operationalized with five main categories that may additively be related to each other (housing, work, interpersonal relationships/social support, finance, and leisure time), would predict outpatient treatment outcome. We assumed that these variables should significantly contribute to an outpatient treatment outcome model even if static risk and (some) dynamic risk factors (previous delinquency and incarceration, index offence, psychiatric diagnosis etc.) are accounted for.

## Materials and Methods

### Sample

The dataset includes *all* patients who had been referred to forensic outpatient treatment in a Baden-Württemberg forensic psychiatric clinic between 2015 and 2017. For this analysis, datasets from N = 391 patients were used. Of these, n = 71 cases (64 men, 7 women) had been discharged from outpatient treatment according to the criteria of discharge that apply in Baden-Württemberg forensic psychiatric units. These include regular discharge, crisis intervention in relation to acute psychiatric symptoms or deterioration of the legal prognosis (§ 67 h, German Legal Code), imprisonment or back-referral to regular treatment), termination of the parole, change of residency, revocation of the suspended measure (§ 67g, German Legal Code), and death.

In n = 3 cases, outpatient treatment had been ordered without prior (regular) inpatient treatment. These cases did not compare with all others in the sample and were therefore excluded.

As opposed to the men, all women had finished their therapy regularly. In order to rule out possibly misleading gender effects, the seven women were excluded from the current analysis.

Thus, the final sample resulted in n = 61 cases. These patients were m = 37.07 (SD = 9.81) years old when admitted to outpatient treatment units. At the time of their first conviction, they were m = 23.6 (SD = 7.45) years old. When admitted to the Baden-Württemberg forensic psychiatric system, they had m = 4.97 (SD = 5.5) entries in the German police register. N = 47 (77%) individuals had a school leaving certificate, n = 26 (43%) had a professional qualification and n = 20 (33%) had a migration background.

Favorable outcome was defined as a regular discharge from forensic outpatient treatment, and compared with all other types of discharge types not associated with successful treatment. Two groups were formed. Regular discharge and end of parole were considered successful treatments (group one with n = 25). Crisis intervention/limited order for measure taking effect (German legal code section 67 h), revocation of suspended measure/conditional release, (German legal code section 67g), and imprisonment or forensic inpatient treatment were considered as unfavorable outcomes (group two with n = 36). Among group two, six individuals were reconvicted in relation to property, traffic, and drug offences, but no-one for violent offences. Short term imprisonment was ruled in five cases, one prison sentence was suspended. Re-hospitalization typically occurred in relation with a crisis intervention due to violations of the court orders underlying conditional release.

### Methods

Since 2014, all forensic outpatients associated with forensic psychiatric units in the Federal State of Baden-Württemberg have been evaluated. A computer-based assessment tool on personal and treatment process variables is used. Data are gathered on an annual basis (reporting date, 31^st^ December), comprising key information on the preceding inpatient treatment: For the present study, we used complete data from three calendar years (2015, 2016, 2017), focusing on the following epistemological domains:

*Personal variables*, e.g. legal basis of inpatient treatment, school and professional qualifications, work, and migration history/migration background;

*Clinical assessment data*, e.g. the main diagnosis/main diagnostic group, psychiatric, and forensic history of the patient and history of substance abuse; and

*Legal criminological data*, e.g. the number of legal convictions prior to admission to a forensic psychiatric hospital, age at first documented delinquency, age at admission to outpatient treatment, duration of previous prison sentences, total duration of inpatient treatment, work time until admission to forensic psychiatric treatment, and the type of index offence.

The assessment tool also contains information on a patient´s current legal and parole status, his/her current living conditions including work and social situation, information on the professional network assigned to help the client in the outpatient setting, client behavior (treatment compliance), and relapse and re-offences (22. For the present study, we analyzed the following variables^1^:

(1) housing: independent housing, sheltered housing, and homelessness or otherwise instable living conditions; (2) work: regular work, assisted work, and no day structure; (3) relationships: stable vs instable relationship/partnership or no relationship at all; supportive familial and non-familial social networks vs. unstable or no social network at all (4) money: satisfactory versus deficient money management; (5): leisure: supportive versus problematic leisure activities.

To ensure the validity and reliability of the tool, all entries (categories, sub-categories, single variables) are explained in a glossary accessible to all forensic therapists working in forensic psychiatric units across the State of Baden-Württemberg. The glossary has detailed instructions on the meaning and content of the items, guiding data-managers through otherwise difficult to rate items. This is to make sure that therapists understand the same thing by each variable. The data were entered by the patient's principal therapist. Entries were electronically checked for plausibility and consistence. Additionally, manual checks were carried out. All data sheets were validated and finally approved of by each departments' Chief Medical Officers. The protocol requires that before release for documentation and research purposes, the data must have been validated by at least three professionals from different professional domains (psychologists, data managers, medical officers).

Thus, no researcher was or has been able to identify individual patients using the dataset. The data was collected and computed in accordance with the data protection requirements set out in the EU General Data Protection Regulation (Regulation EU 2016/679), the German federal data protection act (Bundesdatenschutzgesetz), and the data protection act of Baden-Württemberg including a special law for the mentally ill. These laws regulate the circumstances under which personal data may be used i.e. for research purposes or other purposes that may supersede the interests of an individual not to disclose personal data. Before conducting this research, the data have been anonymized to the researchers.

### Data Analysis

For categorical variables, *Chi^2^* or Fisher’s exact tests were used. For one-way group comparisons of continuous variables, Mann-Whitney-U tests were used (previous work on the data showed that some variables did not meet the pre-conditions for parametric analysis).

In order to investigate the contribution of the variables on favorable, respectively unfavorable outcomes, a logistic regression model was calculated.

Analyses were performed with IBM SPSS statistics (version 25) and R.

## Results

### Descriptive Analysis

The groups significantly differed on the type of index offences (*z* = 11.11, *df =* 2, *p* = .004, Cramer's *V* = .43). They also differed with respect to a person´s history of migration, with migrants having poorer outcomes, i.e. a higher risk of assignment to group two (unfavorable outcome) than non-migrants [in group two, more patients had a migration background (z = 5.42, *df* = 1, *p* = .027, Cramers *V* = .30)]. Further analysis showed that the finding is not due to migrant/non-migrant differences in the distribution of their main offences (χ*^2^* = 225, df = 2, ns); neither is it directly related to the patient´s living situation while in outpatient treatment (χ*^2^* = 4.06, df = 2, ns). Yet, in-depth analyses of the figures suggested a tendency for poorer outcomes (i.e. back-referral to inpatient care, revocation of conditional release) for individuals with a migration background who, during outpatient treatment, lived either alone, or with their family. Given that an person lived in alone or in the family of origin, his relative risk to be a migrant when conditional release was revoked, was RR = 2.0, OR = 6.0 (Living alone/family of origin, poor outcome: migrants, n = 8, non-migrants n = 4; Living alone/family of origin, favorable outcome: migrants n = 2, non-migrants n = 6). Migrants also tended to be less likely than non-migrants to receive professional assistance in some type of community based residential facility (i.e. psychiatric nursing home, outpatient assisted living, resettlement home). Given that a person lived in a community based residential facility, his relative risk to be a migrant when conditional release was revoked, was RR = 1.69, OR = 4.08 (residential care, poor outcome: migrants, n = 7, non-migrants n = 12; residential care, favorable outcome: migrants n = 2, non-migrants n = 14).

There were no significant group differences with respect to diagnostic group, mental illness or alcohol or drug dependency (a treatment according to § 63 of the German penal code is related to mental illness while the treatment according to § 64 is primarily related to substance abuse^2^), or medical compliance. Having a history of substance abuse by the time of the index offense failed to reach significance.

**Table 1** has a full description of relevant categorical variables per group.

**Table 1:** Categorical actuarial variables by outcome group; p and effect size.

	Patient group						
	One: Regular discharge		Two: Unfavorable outcome		Significance level		Effect Size
	n	*%*	n	*%*	Chi^2^ (df)	p	Cramer's V
Main offense*	25	*100*	36	*100*	11.11 (2)	.004	.427
(Attempted) Killing of a person	10	*40*	2	*6*			
Violent offense^1^	11	*44*	24	*67*			
Other offense^2^	4	*16*	10	*28*			
Legal basis of inpatient treatment	25	*100*	36	*100*	.05 (1)	1.00	
Section 63	21	*84*	31	*86*			
Section 64	4	*16*	5	*14*			
Diagnostic group	25	*100*	36	*100*	.07 (1)	1.00	
Psychotic disorders	18	*72*	27	*75*			
Other disorders^3^	7	*28*	9	*25*			
Migration background*	25	*100*	36	*100*	5.42 (1)	.027	.298
yes	4	*16*	16	*44*			
History of substance abuse	25	*100*	36	*100*	4.18 (1)	.062	
Yes	6	*24*	18	*50*			
Medical compliance	20	*100*	30	*100*	3.13 (1)	.140	
Compliance problems	5	*25*	15	*50*			

When more than one cell contained less than five cases, exact Fisher-Tests and z-values were calculated. *Indicates statistical significance (p < .05).

1 included assault and other violent offenses.

2 Included sexual offenses against adults or minors, theft, arson, and other offenses not specified in the original data due to low base rates.

3 included personality disorders, sexual preference disorders, substance related disorders, affective disorders, and mental disability.

**Table 2** shows one-way group comparisons of relevant continuous variables. None of these variables significantly differed between the two groups. Equal mean ages at first documented delinquency, equal mean number of entries in the German police register, and equal mean total durations of prior prison sentences suggest that the two groups did not *a priori* differ in criminal risk.

**Table 2:** Continuous actuarial variables by outcome group; p and effect size.

	Patient group					
	One: Regular discharge		Two: Unfavorable outcome		Significance level	
	n	Mean (SD)	n	Mean (SD)	z	p
Age at first documented delinquency	25	23.80 (7.43)	35	23.46 (7.57)	-.23	.826
Age at admission to outpatient treatment	25	37.72 (10.94)	36	36.61 (9.08)	-.11	.916
Number of entries in German police register	25	5.64 (6.06)	36	4.50 (5.13)	-.59	.560
Mean total duration of prior prison sentences (months)	25	17.60 (35.33)	36	14.25 (31.93)	-.56	.585
Mean total duration of inpatient treatment	25	72.76 (50.64)	36	80.86 (51.54)	-.80	.429
Total work time until inpatient admission (months)	25	69.16 (69.25)	36	66.75 (79.56)	-.70	.490

**Table 3** has the results on variables considered important for outpatient forensic treatment. Prosocial leisure activities and the quality of an individual´s social network differed significantly between the groups, but there are other figures calling for a close look into the sub-categories of the living or the work situation. Hence, we included all variables reported in **Table 3** into the regression analysis.

**Table 3:** Outpatient outcome variables by outcome group, p and effect size.

	Patient group						
	One: Regular discharge		Two: Unfavorable outcome		Significance level		Effect Size
	n	*%*	n	%	Chi^2^ (df)	p	Cramer's V
Living situation	25	*100*	36	*100*	1.80 (2)	.476	
Homelessness	1	*4*	5	*14*			
Sheltered living	16	*64*	19	*53*			
Independent living	8	*32*	12	*33*			
Working situation	25	*100*	36	*100*	3.94 (2)	.148	
None	5	*20*	14	*39*			
Sheltered work	10	*40*	15	*42*			
Regular work	10	*40*	7	*19*			
Stable relationship	25	*100*	36	*100*	.05 (1)	1.00	
yes*	3	*12*	5	*14*			
social network	25	*100*	36	*100*	4.45 (1)	.035	.27
Insufficient social network**	13	*52*	28	*78*			
Money management	25	*100*	34	*100*	3.74 (1)	.094	
Poor money management	5	*20*	15	*44*			
Leisure activities	24	*100*	36	*100*	7.51 (1)	.008	.35
Prosocial leisure*** activities	11	*46*	5	*14*			

*A relationship was coded stable if “firm stabilizing partnership” was marked in the glossary.

**A social network was regarded “insufficient” if contacts with family members or extra-familial contacts were regarded problematic or destabilizing according to the glossary, and if “social withdrawal/loneliness” was marked.

***Indicates statistical significance (p < .05). Leisure activities were defined pro-social, if a patient was rated “independent problem-free leisure time” or “unproblematic recreational activities under supervision” according to the glossary.

### Logistic Regression Model

Both all variables testing significant after univariate analysis and those describing probands' living conditions were entered into a logistic regression model. These included the type of index offence (main offence) and migration status (**Table 1**), living situation, working situation, (stable) relationship, social network, and money management (**Table 3**). In order to rule out multi-collinearity, variance inflation factors (vif) and tolerance (1/vif) were calculated for each variable. The values were within the limits recommended in the literature (10, or 0.1 respectively). Residuals were analyzed with respect to outliers. There were none. Based on these figures, we considered the requirements for the calculation of a logistic regression analysis to be fulfilled.

The variables were entered stepwise, starting with main offence, and migration background. Two variables predicted group membership. The resulting model was significant, χ^2^ (2, *N* = 49) = 15.61, *p* < .001), explaining 32% (*R* ^2^ by Nagelkerke) of the variance. **Table 4** displays the details.

**Table 4:** Logistic regression analysis.

	Significance level						95% CI for Exp (b)	
	b	SE	Wald	df	p	Exp (b)	Lower	Upper
Prosocial leisure activities	2.13	.77	7.78	1	.005	8.45	1.89	37.85
Migration	-1.80	.75	5.74	1	.017	.165	.038	.721
Constant	-.367	.37	.970	1	.325	.693		

All descriptive variables that differed significantly between the two groups as well as all variables indicating the living situation of the patient were entered into the logistic regression model. The reference category was regular discharge (favorable outcome). The stepwise enter method was used based on a conditional selection, starting with the main offence, and migration background. The analysis resulted in two variables that significantly predicted group membership. 72% of the patients were correctly assigned to the groups using these two variables. Nagelkerkes R-Square of.318 indicated that 31.8% of the variance in the data can be explained using this model.

## Discussion

### Epidemiological Findings

Most patients who committed or attempted homicide ended therapy in a regular manner. This could be due to the fact that according to the RNR-principle (e.g. 23, 24), more resources had been allocated to the treatment of high risk offenders. It is also clear that homicides and attempted homicides are offences associated with a relatively low base rate (25, 26); yet, individuals who committed these offences tend to be treated for above average periods of time. Ross and colleagues (27) explored the patient characteristics in an inpatient sample and found that having committed a sexual or lethal offense was associated with higher odds of being a long-stay patient during inpatient forensic treatment. In our sample however, the mean duration of inpatient treatment prior to conditional release did not differ between the groups, indicating that the duration of inpatient treatment is not critical once a patient is deemed fit for release. Rather than treatment duration, particular types of index offences leading to initial inpatient treatment (i.e. those with relatively high base rates; violent assaults and other violent offences; some sexual offences associated with hands-on violence) seem to be associated with unfavorable outcomes of outpatient treatment.

The legal basis of inpatient treatment (sections 63 and 64 German Penal Code) and main diagnoses at the time of admission to outpatient treatment did not distinguish between the two outcome groups. Whether or not a patient had a history of substance abuse and the patient's level of medical compliance also failed to reach statistical significance. All variables reflecting a patient´s criminal history (**Table 2**) did not significantly distinguish between the groups, which is not what we should have expected based on the findings of Eisenberg et al. (5) (criminal history counts among the central eight and is generally related with treatment outcome). Comparing the findings of our study with the evidence put forward by Eisenberg et al. (5), we believe that our sample was much more homogeneous with respect to the central eight fed into Eisenberg´s analysis. Furthermore, our data stem from one single German federal state, Eisenberg´s data comes from several Western countries; only studies in which community sentencing was operationalized as an imposed outpatient/community-based treatment (such as a psychological or addiction treatment, probation, or supervision) were chosen for inclusion. Finally, and most importantly, outcome was defined differently between the studies. In contrast with Eisenberg et al., we predicted adherence to and regular termination of outpatient treatment, not primarily criminal recidivism. Substance abuse is usually expected to be a predictor of poor outcome (7–9) In our study, it did not. Taking into account that the sample had spent an average of 74 months in inpatient treatment, this is remarkable. Of course, forensic inpatient treatment targets drug and alcohol addiction as one of the main treatment goals, but substance addiction obviously continues to have long-term effects on a patient´s chance to successfully pass outpatient treatment. The majority of outpatients received a court order not to consume alcohol and illicit drugs. It may be that these orders took effect and probation officers and others involved in the outpatient care system did their job very well, helping to prevent substance-addicted individuals to keep away from the drugs. A less optimistic view pertains to a statistical argument: a history of substance abuse was clearly more prevalent in the poor outcome group (50% vs. 24%). Yet, the comparison failed statistical significance (p = .062, **Table 1**). Larger samples might have produced a different result.

Previous analyses yielded strong effects of relationship status (marriage) (13) and other turning points in a general offender populations. In our study, we could not replicate the findings in the literature. It is worth noting, however, that we did not measure the formal status of a relationship, but relationship stability. Relationships can be stable (or unstable) regardless of whether one is married or not. Only few individuals in both groups had firm and stable relationships by the definition the glossary. Following a long time of intensive psychiatric care, it may be difficult to start, or to maintain a stable (intimate) relationship in this sense. Göbbels et al. (18, 28) noted that the significance of relationships on forensic outcomes may depend on whether or not a person has a history of mental illness. The obstacles to re-enter society, to find and keep a job and to cultivate contacts with friends and families may be different and even more difficult in mentally ill offenders compared with prisoners. Stable intimate relationships in our samples were generally rare, which may be another reason why this variable failed to reach statistical significance.

Being a migrant was associated with a higher chance of assignment to group two (unfavorable outcome). Given that a person lived alone or in his family of origin, the relative risk of this person to be a migrant (whose conditional release has been revoked) was elevated. Migrants also tended to be less likely than non-migrants to receive professional assistance in some type of community based residential facility (i.e. psychiatric nursing home, outpatient assisted living, resettlement home).

All of these figures are rather small, which is why the following conclusion remains somewhat speculative: it may be beneficial for forensic outpatients to live an environment that provides regular and qualified professional care rather than settings, where this kind of support is not provided [i.e. in some families of origin (15)]. Placement of patients in families of origin living in socially disorganized neighborhoods (16) may also be associated with unfavorable outcomes.

Stable (pro)social networks including have been described as important pillars of successful reintegration of forensic outpatients into society (e.g. Smith et al. (15), 18, 28). In our study, similar findings underline the significance of social networks in helping patients organize their lives in freedom. From a clinical point of view, this is not easy to accomplish. Most patients experience many years of intensive in-patient treatment before outpatient treatment is considered, and they need to adapt to the various challenges that come with life in the society. Hence, continuous social support provided by (pro-)social networks (family, peers, social workers, and other professionals) may be regarded an inevitable precondition for patients to succeed in the long term.

### Logistic Regression

Two significant predictors of outcome were identified. Pro-social leisure activities seem to be the most important predictor of long-term outcomes in forensic outpatient treatment in the German Federal State of Baden-Württemberg. Leisure time is generally referred to as time spent away from business, work, domestic work, and education, as well as necessary activities such as eating and sleeping. Leisure time may be associated with the notion of freedom: the freedom to do what one wants to do. If a patient is able to fill the free time (which probably amounts to several hours a day) with activities that are not associated with criminogenic needs and recidivism (e.g. alcohol and other substance abuse), and/or help the patient to develop a sense of meaning in what he is doing, he will be less likely to fail in outpatient treatment. It is worth noting that leisure activities appear to be more important than general housing conditions, offence patterns, medical compliance, relationships, and social networks. But of course, these variables are inter-correlated, suggesting that a patient's ability to spend leisure time in a pro-social way cannot be viewed independently of these variables. In our view, pro-social leisure activities should be conceived of as a meaningful correlate of the variables entered into this model, entailing many aspects of the above-mentioned variables normally thought to be essential for providing successful forensic after-care. This may be why these variables did not significantly contribute to the regression model.

Migration is the second statistically significant predictor of outcome. Non-migrants appear to do better than migrants. The reason for this finding may be associated with the fact that migrants are less likely to receive ongoing professional support other than the services provided by forensic ambulances and probation personnel. It may be that many patients in outpatient settings profit from some type of sheltered living after discharge from forensic psychiatric inpatient treatment, but migrants are less likely to live in a sheltered environment.

To summarize, the results indicate that after release from inpatient treatment, pro-social leisure activities may be crucial for the patients' chances to succeed. Professional support in a protected environment appears to be more important for the outcome than any set of actuarial variables except for pro-social leisure activities (type of index offence, number of prison sentences, age at first delinquency etc.), or clinical and risk management variables comprising diagnostic group, (medical) compliance, relationship quality, money management, and supportive social networks. While it may be beneficial for outpatients to receive additional support in a professional housing environment, migrants are less likely than non-migrants to live in sheltered environments, and if so, they do not receive the same amount of additional services helping them to structure their daily life activities. This conclusion is somewhat speculative as the figures supporting this notion are rather small. To substantiate this claim, more data are needed.

### Limitations

There is a limitation related to sample size. Outpatient data assessment in Baden-Württemberg forensic psychiatric outpatient units started in 2015, but the number of patients released from outpatient treatment until the end of 2017 was still relatively small. Taking this into account, the results should be considered preliminary. Some tendencies showed but failed to reach statistical significance (i.e. history of substance abuse, working situation, money management, and medical compliance). In order not to reduce statistical power to the effect that statistical trends emerging from relatively small samples cannot be observed at all, we did not adjust p levels to multiple comparisons. Larger samples will yield more stable results in future studies on this matter and they will help to resolve open questions as regards the role of migration status for the prediction of outcome.

## Data Availability Statement

The datasets generated for this study are available on request to the corresponding author.

## Ethics Statement

Ethical review and approval was not required for the study on human participants in accordance with the local legislation and institutional requirements. Written informed consent for participation was not required for this study in accordance with the national legislation and the institutional requirements.

## Author Contributions

KK, TR, and JB planned the research project in equal parts with regard to aim, methodology, and hypotheses. The authors regularly discussed the research process. The authors contributed as follows: KK did the literature search, conducted part of the calculations, and wrote most of the first draft. TR intensively revised the first draft. JB developed the initial idea, did part of the calculations and writings of the first draft and the final revision.

## Conflict of Interest

The authors declare that the research was conducted in the absence of any commercial or financial relationships that could be construed as a potential conflict of interest.

## Supplementary Material

The Supplementary Material for this article can be found online at: https://www.frontiersin.org/articles/10.3389/fpsyt.2020.00042/full#supplementary-material

## Footnotes

1 Details of core variables investigated in this study are listed in the glossary (see **Supplementary Material**).

2 Forensic outpatient treatment in Baden-Württemberg focuses primarily on patients released from a forensic hospital in the context of a hospital treatment order according to section 63 of the German Penal Code. Patients released from addiction treatment according to section 64 are only admitted to outpatient treatment if they have serious comorbid psychiatric disorders. In other Federal States, the regulations may be different resulting in a different distribution of patients amenable to outpatient treatment.
