# Supplementary material for: Forensic Outpatient Variables That May Help to Prevent Further Detention
Source: Front Psychiatry. 2020 Feb 13;11:42. doi: 10.3389/fpsyt.2020.00042 (PMC7031275; doi:10.3389/fpsyt.2020.00042)
Supplement: Supplementary file 1 [file DataSheet_1.docx]

**Appendix**

Glossary

**Migration background**

Here it must be determined whether the subject has a migration background or not. For migrants, the definition of the Federal Statistical Office applies (see selection menu).

Definition: Migrant, late repatriates, and foreigners

People with a migration background or migrants are people who were not born in their respective country of residence (here: Germany), and their descendants.

The category *immigrant foreigners* *with residence permits* includes immigrant EU foreigners who do not need a residence permit. These individuals are granted unrestricted residence status under current EU law, which is equivalent to a formal residence permit.

Aussiedler (repatriates) or Spätaussiedler (late repatriates) are all ethnic German immigrants to Germany. The German heritage must be documented by appropriate identification documents. The definition according to § 4 of the law on displaced persons and refugees (Spätaussiedler, http://bundesrecht.juris.de/bvfg/__4.html) applies.

*Foreigners* are grouped as follows:

- A "foreigner born in Germany" was born in Germany, but has no German passport.
- A "naturalized foreigner" is a foreigner with a German passport, regardless of whether he was born in Germany or not.

Asylees and asylum seekers are classified under the categories "immigrant foreigners with or without a residence permit".

The country of origin can be selected in the electronic sheet.

**Medical compliance**

From a forensic psychiatric point of view, this item mainly refers to what is defined as "necessary medication". These will mainly be antipsychotic medications or phase prophylactics, which both have a beneficial effect on the symptoms and the course of the psychiatric illness. Handling drug-suppressing medication also falls into this category. If the medication is subject to a directive, it is automatically considered "necessary".

"Recommended psychopharmacological treatment" is not directly related to dangerousness and legal prognosis. It is recommended to relieve symptoms and to support psychosocial treatment.

*Good compliance*: On behalf of the patient, there is insight into the necessity of treatment. As far as possible, the patient will seek joint treatment planning with the doctor. He voices possible side effects the medication and actively seeks solutions of problems.

Not necessarily indicated, but recommended treatment is discussed with the practitioner in the sense of "informed consent" and, if agreed, also taken as discussed.

*Mostly compliant:* The necessary medication is usually taken as prescribed (confirmed by the clinic and TDM). Insight into the necessity of treatment is fluctuating and / or predominantly motivated by others (not by the patient himself). Rarely does he take the opportunity (organizational, personal difficulties, holidays of important caregivers, etc.) not to take the medication. This category should also be marked if regular medication is provided with appropriate human and organizational measures (for example in intensive care homes).

*Unreliable medical compliance:* The clinic and TDM raise doubts about the patient´s use of medication. The patient makes no secret of his refusal to take the medicine. Under pressure and appropriate interventions in case of infringements of instructions, the medication is eventually taken. But normally, the patient uses every opportunity to not take the medication. The intake is rejected, but there is no consistent pattern of refusal.

*Denial of medication:* The patient clearly rejects or refuses medication for at least one month.

**(Stable) relationship**

*Partnership:*

*No partnership:* The patient claims to have neither a partnership nor "fleeting sexual contacts"

*Firm stabilizing partnership:* a solid partnership is an interpersonal relationship that should include at least some of the following dimensions: sexual contacts, emotional familiarity, shared life planning, shared housing, raising children, marriage / life partnership. An important criterion is the assumption of responsibility on the part of the patient. Included are homosexual or heterosexual relationships of at least 2 months duration that do not break the law (i.e a sexual and emotional relationship of an 18-year-old to a thirteen-year-old is not included).

*Fleeting sexual contacts:* non-binding, mainly intercourse-only contacts, one-night-stands, contacts with prostitutes, etc.

*Short but unproblematic partnerships*: relationships that go beyond sexual intercourse, but are terminated / changed after a short period of time. The separations do not lead to a lasting destabilization of the patient.

An exception is the relationship pattern described in Item 17 of the PCL-R ("many short marriage-like relationships"). These are relationships that are mostly related to spatial coexistence and appear to have a long-term perspective (view the description of PCL-R Item 17). Patients before the age of 30 who have at least two, and after the age of 30 at least three such partnerships fall into this category. This relationship pattern is considered problematic. An essential criterion is that the relationships appear to be interchangeable. In this case, also specify the following relationship pattern:

Conflicting / destabilizing partnership: this includes an unstable pattern of relationships that repeatedly results in significant clinical deterioration, sustains a mental disorder, or is a symptom of the disorder ("many short marriage-like relationships"). Relationships with unfavorable effects on legal prediction (see Item 76), or solely functional relationships (see above) are also included.

**Social network**

Classification should be preceded by an analysis of individual risk and protective factors. The following aspects can be taken as the basis for the assessment:

*Stabilizing contacts and structures:* these contribute to mental stability helping to reduce symptoms of illness and the consequences of mental disability; they may also promote psychological well-being and enhance "quality of life". Abstinence and a prosocial lifestyle are encouraged. They are fundamentally positive about psychiatric treatment and forensic care.

*Destabilizing or problematic structures and contacts:* mental and somatic health is put in danger. Social contacts support a criminal lifestyle, or prevent more a prosocial development. Contacts encourage substance use and other infractions of the law. By and large, the patient's social contacts are opposed to forensic aftercare and psychiatric treatment.

**Extra-familial social contacts**

*Little social contact but not dissatisfied:* the patient has only occasional "superficial" contacts with other people; he appears fine. This situation is not considered to be a risk factor: e.g. Patient with schizoid personality disorder, who pursues a full-time job; except for peer-to-peer and occasional contacts with neighbors, he lives in seclusion.

*Social withdrawal / loneliness* as a risk factor: this can pose a risk at various levels:

- The symptoms (for example, negative symptoms) are partly due to a lack of social contacts.
- This leads to serious malfunctions: the patient feels neglected
- The patient suffers from these
- The legal prognosis worsens: pedophile patient who spends his leisure time on the computer.

This category should also be marked if a patient does not accept or refuses offers made by the supervising social-psychiatric institutions.

*Social contacts promoted by an institution*: the patient participates in organized activities of the social-psychiatric care system. Occasional non-organized contacts with individuals met in these activities fall into this category. If the patient met someone through activities organized by social psychiatry, but now meets them independently of the social-psychiatric care system (in order to spend time together), the category "*predominantly stabilizing contacts with friends / acquaintances”* should be selected.

*Other:* e.g. regular online contacts that are experienced as non-problematic and rather stabilizing.

**Contacts with family members**

*Sporadic but unproblematic contact:* rather non-binding contact, in which support plays a minor role.

*Regular stabilizing contact:* Whether a contact is considered to be regular must be deduced from the overall context (for example, close relatives vs distant relatives). It is important that there is a certain emotional involvement or relationship of trust, and that the patient receives material and / or emotional support.

*Problematic / destabilizing contacts:* this may include, for example, close and, at first sight, stable contacts with, for example, parents; the criterion is that the contact results in a dysfunctional or dependence-promoting relationship with the patient.

**Living situation at discharge (housing) and changes in housing**

*Resettlement home:* this is a dormitory financed by reintegration benefits (SGB IX, XI, XII). The goal is psychosocial rehabilitation with preparation for a less supervised form of living.

*Psychiatric nursing home:* the home is financed according to SGB XI, XII. The inhabitants usually live there permanently.

*Closed dorm:* a closed psychiatric nursing home

*Other types of homes (e.g. homes for ex-prisoners):* Homes that either finance themselves through "help to overcome particular social difficulties" according to SGB XII or other financial means (club funds, etc.). This also includes

*Outpatient assisted living:* the financing according to § 55 (2) no. 6 SGB IX

*Foster family:* the family concerned receives support and benefits according to SGB VIII Youth Services, SGB IX / XII integration assistance

The above categories are referred to as “Sheltered living”.

*Homelessness:* Homelessness is defined as living in housing that is below the minimum standard or lacks secure tenure. People can be categorized as homeless if they are: living on the streets; moving between temporary shelters, including houses of friends, family and emergency accommodation; living in private boarding houses without a private bathroom and/or security of tenure.

*Changes:* the housing situation at the end of the respective reporting period is based on the comparison of the housing situation documented in the previous survey.

**Work and Employment**

This item focuses on the psychosocial level of functioning and the nature and extent of day structuring. Income is included under Item 79 "Financial Situation".

1. Labor market: This is a job subject to social security contributions or employment based on supply and demand in the labor market.

2. Labor market: government-funded job.

*Self-employment* is assigned to the 1st or 2nd job market depending on the availability of funding.

*Education / Training:* Education and training measures not based on benefits for participation in working life in accordance with SGB IX Chapter 5^[[1]](#footnote-1)^. Education/training should lead to a specific occupation-related qualification.

*Vocational rehabilitation:* these are financed by benefits for participation in working life according to SGB IX Chapter 5.

*Sheltered workplace:* the workplace is offered as part of special facilities for disabled people: workshops for the disabled (Book 9 of SGB IX). *Integration projects:* integration departments, companies, companies acc. Chapter 11 SGB XI).

*Work therapy:* this is provided by the GKV (§ 42 SGB V) or "as a low-threshold job offer" by social assistance.

*Social psychiatric day-structuring measures:* e.g. psychiatric day care.

*No work / casual work:* work is less than 10h per week.

*Parental leave / activity in the household:* at least one other person lives in the household and is provided with the activity (household management, education, nursing). The time required should average at least 3 hours per day.

*Retirement:* As a result of retirement pension, there is no regular employment.

*No fixed day structure:* less than 10 hours per week. Alternatively: less than 15h in one of the other possibilities for day structuring.

In the event that the work situation is not clearly mapped by any item, the most appropriate item is chosen.

**Money management**

The item evaluates the patient´s economic, legal and social problems caused by his behavior, i.e. through his handling of money.

*No:* the patient handles his money well, i. e. all essential obligations and financial needs can be met. If debts are made, their purpose is traceable and compatible with the patient´s economic performance. When a supervisor is involved and the patient co-operates well in financial matters, this item should also be marked.

*Yes:* money management is difficult in various ways: expenses clearly exceed income; the patient is resorting to illegal, "semi-legal" or other problematic means (working in the red-light milieu) to increase his income; In order to increase his income, he works too much (secondary jobs, etc.); interpersonal relationships suffer due to financial issues.

Not applicable: the patient is not free in financial matters (this is the case, for example, in some intellectually disabled patients).

**Leisure activities:**

*Independent problem-free leisure time:* Leisure is self-determined according to the patient´s needs.

*Unproblematic recreational activities under supervision:* the patient participates in recreational activities offered by the social psychiatric care system.

*Problematic leisure activities:* These can be seen as problematic in various respects:

- The activity is associated with individuals and structures considered destabilizing in the preceding items.
- The activity promotes a dynamic which is considered problematic, e.g. Red light milieu / SM scene in sex offenders, or practicing full-contact martial arts after having committed a violent offence.
- The activity allows access to potential victims or risks, e.g. leading youth groups through pedophile offenders, access to firearms in the shooting club
- The activity is almost exclusively carried out without contact with other people (i.e. anonymously), which is considered a destabilizing factor: excessive Internet use
- The activity precludes a balanced and health-promoting lifestyle: it leads to significant physical or physical overload, endangers family structures, or makes it impossible to balance work and relaxation.

*Lack of leisure time:* this can have different causes (lack of interest, lack of financial and real opportunities, way of life)

1. SGB IX chapter five refers to the German Social Law. [↑](#footnote-ref-1)
